# Living Alone: Associations with Diet and Health in the Spanish Young Adult Population

**DOI:** 10.3390/nu15112516

**Published:** 2023-05-29

**Authors:** Elena Sandri, Marcelino Pérez-Bermejo, Asensi Cabo, Germán Cerdá-Olmedo

**Affiliations:** 1 Doctoral School, Catholic University of Valencia San Vicente Mártir, c/Quevedo 2, 46001 Valencia, Spain; 2 Faculty of Medicine and Health Sciences, Catholic University of Valencia San Vicente Mártir, c/Quevedo 2, 46001 Valencia, Spain; 3 SONEV Research Group, Faculty of Medicine and Health Sciences, Catholic University of Valencia San Vicente Mártir, c/Quevedo 2, 46001 Valencia, Spain

**Keywords:** healthy eating index for the Spanish population, frequency of food consumption, eating at home, eating alone, living alone

## Abstract

Eating together as a family has important health benefits, as the diet is more balanced and of a higher quality. Eating together is also a factor in the prevention of diet-related diseases. The promotion of family and shared meals is currently a public health strategy. The aim of this research was to study the eating habits of the Spanish young adult population and their impact on health. An observational, descriptive, cross-sectional study was carried out using surveys. A questionnaire was designed and validated to explore a set of variables related to food and health. The dissemination was carried out through social networks by means of an online form, using non-probabilistic snowball sampling to obtain a sample of 17,969 subjects aged between 18 and 45 years. We found statistically significant differences between people living in a family home compared to people living outside a family home in the healthy eating index for the Spanish population, fish consumption, and fried food consumption. This suggests that the nutrition of people living in a family home is healthier, although their BMI is higher. People living together have a statistically higher healthy eating index value than people living alone; consume fast food, fried food, and ultra-processed food less frequently; and eat fish more often. On the other hand, people who live in a family home or are accompanied are more likely to have a sedentary lifestyle and are less physically active. It was concluded that people living alone have a worse healthy eating index than those living with company, which seems to indicate that nutritional interventions should pay attention to people living alone as a variable to be taken into account in future analyses.

## 1. Introduction

The importance attributed to proper nutrition is becoming increasingly evident, both from a social point of view and from the point of view of improving health. Proper nutrition can help avoid the negative consequences that an incorrect diet can cause, such as cardiovascular diseases [1], some cancers [2], and obesity [3], among others. There are different indices and indicators in the literature that allow us to study types of diets and their impact on health, including indicators of diet quality related to food groups [4,5,6] and indices of adherence to a particular dietary pattern [7].

A growing phenomenon that has become a major public health problem in recent years is the loneliness experienced by many individuals, particularly in Western society. Social isolation has been shown to be an important risk factor for the development of different pathologies such as obesity [8], depression [9], and dementia [10], among others.

Previous studies have shown that living alone influences eating habits, as people who are not accompanied by the community find it difficult to maintain a healthy diet due to factors such as social isolation and a lack of support [11]. Eating together is a fundamental part of social life, as eating plays an important role in people’s lives [12,13]. 

Loneliness is a growing public health problem among young people, and it is associated with poor physical and mental health, poor educational outcomes, and poor personal well-being [14,15,16]. There are a number of socioecological factors associated with loneliness, including sociodemographic, social, health and well-being, and community factors [15,16]. The geographic region may account for differences in loneliness, and initiatives at the local level may be best suited to combat loneliness [15]. Interventions have been shown to reduce loneliness among youth, but they often target youth that are considered at risk and rarely target youth who report feeling lonely [14]. There is a need for innovation in the treatment of depression, especially among young people, as treatments rarely target loneliness and living alone, which are key risk factors in the onset, maintenance, and development of depression [17]. In young people, eating is also affected by loneliness, and the recent COVID-19 pandemic has highlighted this [18]. In general, young people are a special group experiencing a double transition from living with their parents to living on their own, and many of them start to acquire or enhance unhealthy eating habits [17].

From the above considerations, it can be inferred that it is not only the type of food an individual eats, but also where and how he or she eats that is important for his or her health and well-being. It has been widely studied that eating as a family, in addition to the pleasure of sharing time with loved ones, has important health benefits [19,20]. Different studies have shown that the quality of life for people who eat as a family is improved, as their diet is generally more balanced and of a higher quality [21]. Eating as a family is also a preventive factor against diet-related diseases such as obesity and eating disorders [22].

In Western countries, the frenetic pace of life means that family meals are often being replaced by meals alone in front of an electronic device, in the car, or at the computer [23]. Sometimes, this mealtime has been greatly reduced or even eliminated and replaced by snacking between meals, which has a negative impact on people’s health [24].

The promotion of family and shared meals has become a public health strategy in recent years [25]. It is therefore of great importance to understand the behavior of these eating and sharing habits in the population, in order to design effective training and information actions. Understanding them also gives us a fairly realistic idea of the health status of this population.

Some papers have pointed out a lack of studies in the scientific literature that comprehensively address the association between living alone and the dietary intake in older adults, as well as the need to further investigate the links between living alone and eating patterns, and how this may affect people’s health and nutritional status [26,27]. Studies that analyze the impact of living alone on people’s eating habits and nutrition are needed, as living alone may come to influence people’s food choices, meal frequency, appetite, and diet quality. In addition, longitudinal studies are needed to examine how loneliness and changes in a person’s living-alone status over time may affect people’s nutritional intake and nutritional status.

In view of the above, the aim of this work was to study the impact that living alone may have on eating behaviors and some health-related habits in the young adult population in Spain.

## 2. Materials and Methods

### 2.1. Type of Study and Sampling

A prospective cross-sectional study was conducted on the young adult Spanish population residing in Spain. For this purpose, Spanish people or settled immigrants aged between 18 and 45 years were included. Chronic diseases or temporary situations that could affect their diet were considered exclusion criteria.

### 2.2. Ethical Approval

The study was approved by the Research Ethics Committee of the Catholic University of Valencia (registration number UCV/2019-2020/152). This study complies with the principles set out in the Declaration of Helsinki [28].

### 2.3. Methodology

Data collection was carried out through the dissemination of a self-developed questionnaire consisting of 53 questions, with some on general and sociodemographic data and others on the frequency of the consumption of different food groups and the frequency of the repetition of health-related sporting and social habits.

In order to test the content validity, the developed questionnaire was disseminated to a pilot group of 53 persons. After analyzing the results obtained from the pilot group and collecting their observations, a nominal group of 7 experts in areas related to the research topic was convened to help in the development of the final questionnaire. The seven experts that participated included the following: two psychologists, a nutritionist, a social educator, two family doctors, and a communication professional. The work of this group of experts resulted in the final questionnaire.

The survey was disseminated telematically using the functionalities of the Google Form program by means of non-probabilistic snowball sampling. The channels used for the dissemination were personal social networks (LinkedIn, Twitter, WhatsApp, and Facebook) and mailing to different national associations and establishments selected for their heterogeneous public (pharmacies, clinics, etc.). The Instagram account @elretonutricional was specifically created to disseminate the survey and contact several professionals and influencers.

The survey was available for online response between August 2020 and November 2021. A total of 22,205 people of all ages and residing in all parts of Spain, including people from the Canary Islands and naturalized foreigners, answered the survey. Surveys that did not meet the inclusion criteria and those with invalid or incomplete data were discarded. Most of the surveys collected were from people under 50 years of age. The explanation for this lies in the fact that the social network mainly used to disseminate the survey was Instagram, and the majority of Instagram users are under 40 years old (63.85%). This is why it was finally decided to limit the sampling to those aged between 18 and 45, where more representative and heterogeneous data had been collected. 

The behavior of the sample was then studied with respect to different health and behavioral variables, differentiating between people living in a family home and those living outside a family home, and also differentiating between people who usually live alone and those who live accompanied. 

### 2.4. Variables

Sociodemographic variables such as sex, age, place of birth and residence, work, level of education, income, and usual residence were collected. Anthropometric and health variables were also collected, such as weight, height, and self-perceived level of health. Finally, data were collected on nutritional habits (consumption of different foods), physical activity, and alcohol consumption.

Some variables were collected in a qualitative format, giving the option of choosing one among multiple options, as is the case, for example, of: sex, place of birth and residence (where a list of all the provinces of Spain was presented), level of education (where a choice was given among all the possible levels of education in the educational system), level of income (where salary steps were presented), usual residence (where a list of possibilities was presented), and all the frequencies of food and drink consumption. Some variables had a continuous quantitative value such as age, weight, and height (which were self-reported values), and others had a discrete quantitative value in the Likert scale format such as a self-reported level of health, quality of sleep, or waking up feeling rested.

The IASE (healthy eating index for the Spanish population) was calculated using a reduced version of the index validated by Norte and Ortiz [29]. This index analyses the frequency of the consumption of foods that are recommended to be consumed daily and weekly, as well as foods for occasional consumption. It also looks at dietary variety, which is essential for healthy eating. A maximum score of 10 on an item indicates compliance with the recommendations proposed by the Spanish Society of Community Nutrition [30] (SENC). The maximum index score is 73.

Self-perceived health was a score on a scale of 1 to 5. The rest of the variables that are not directly contemplated in the IASE, such as the consumption of water, coffee, fast food, fried foods, ultra-processed dishes, fish, and alcohol, as well as sedentary time, were categorized on a Likert scale from 1 to 4 points, as indicated in the Table 1:

### 2.5. Statistical Analysis

Discrete variables are presented as an absolute value and percentage. Continuous variables are expressed as the mean and standard deviation. For the analysis of continuous variables, the non-parametric Mann–Whitney test was used. A two-tailed *p* < 0.05 was considered statistically significant. Data were analyzed using SPSS v.23 (SPSS Inc., Chicago, IL, USA).

## 3. Results

The final sample consisted of 17,969 valid surveys. Table 2 shows the sociodemographic characteristics of the sample.

Table 3 and Table 4 show the behavior of the sample, differentiating between people living in a family home and those living outside a family home, and also between people who usually live alone and those who live accompanied.

With regard to the comparison of the population group living at home or away from home, we found that the BMI of people living in a family home was significantly higher than those living away from home. However, despite having a higher BMI, the healthy eating index was significantly better. We found no statistically significant differences in the consumption of water, coffee, or ultra-processed food. We observed that those living at home consumed alcohol more frequently, were more sedentary, and participated in fewer sports activities than those living away from home. Those living away from home consumed coffee and fried food more frequently and fish less frequently than those living at home.

With respect to the group of people living alone compared to those living with others, we found that there were no differences in their BMI or self-perceived health. On the other hand, it was observed that the IASE values were significantly lower, suggesting dietary impoverishment. They were less sedentary, participated in more sports activities, and consumed alcohol less frequently than those who live together. However, they consumed water, coffee, fast food, fried food, and ultra-processed food more frequently than those living with others. They consumed fish more frequently than those living alone.

## 4. Discussion

The results of this study show that, although the level of self-perceived health was similar in the groups of people living at home and living away from home, the nutritional index was higher in the group of people living at home, which seems to indicate that their food consumption habits were healthier than those of the group of people living away from home. This was corroborated by other variables such as fish consumption, which was more frequent in people living at home. Other studies [31,32,33] have associated more frequent dietary fish consumption (3–4 times per week) with a lower risk of chronic diseases and overall mortality, in particular cardiovascular disease, depression, and liver cancer.

Although the frequency of the consumption of fried food was relatively low for the whole population (between 1 and 2 times a month on average), we found that the consumption of fried foods was less frequent in people living at home than in people living away from home, which is a positive fact from a nutritional and health point of view. On a culinary level, frying food modifies the composition of the food’s nutrients due to different physico-chemical phenomena that take place, causing the appearance of components that are harmful to health such as trans-fatty acids and increasing the number of calories that the food contains due to the absorption of part of the frying fat. For this reason, the frequent consumption of fried foods increases the risk of various diseases such as obesity, type 2 diabetes, hypertension, and cardiovascular disease [34].

In our results, one finding was not consistent with this trend of greater health in the consumption habits of people who eat at home compared to those who eat out, and that was alcohol consumption, which was more frequent among people who eat at home. Alcohol is associated with numerous health problems [35] and has been shown to affect the brain and most organs and systems [36], so it is advisable to limit its consumption in the diet, although its consumption recommendation is ambiguous in most dietary guidelines [37] and some guidelines advise drinking a glass of wine with a meal, arguing that it has cardiovascular benefits [38]. This tendency found in people living at home could be explained by the fact that eating at home favors socialization and conviviality at the table and the adoption of Mediterranean customs, such as drinking a glass of wine with a meal. Although these differences were significant, the average frequency of the consumption of alcohol for all groups was sporadic (around once a week).

Our results show no significant differences in other dietary habits between the group of people living away from home and the group living at home, such as the consumption of water, fast food, and ultra-processed dishes, where the pattern of consumption was similar in both groups. Different studies [39,40] have shown the harmful health effects of fast food and ultra-processed food consumption, such as an increased risk of cardiovascular disease, obesity, and the development of some types of cancer, among others, and it is therefore advisable to limit their consumption. Notwithstanding the differences found, it should be underlined that the frequency of the consumption of fast food and ultra-processed food for all groups was limited (between never and once a month), indicating that we are in the presence of a mostly healthy population.

In terms of sedentary lifestyles and the time spent participating in physical activity, an opposite trend was observed in terms of health. People living in a family home adopted less healthy habits. The degree of sedentary behavior was higher among people living at home compared to those living alone, which has negative consequences for health, as reducing the amount of time spent sitting down can improve an individual’s general health and reduce the risk of obesity [41]. A similar result was found for the time spent participating in sports activities by people living in the family home, which was significantly less than that spent by people living outside the home. This is also a negative aspect for them, because of the health and prevention benefits of regular physical activity for many chronic diseases [42,43]. This point could explain the significantly higher BMI value of people living at home compared to those living away from home, although it may also be related to the age of the sample, as there was a difference of almost 3 years between the two groups. Different studies [44,45,46] have shown that an increasing age of a population leads to an increase in the BMI, which can even result in a doubled BMI over a 10-year period.

With regard to living alone, our results show a lower rate of healthy eating in people who do not live with their families or are unaccompanied, which seems to indicate that their diet is less healthy than that of those living with others, which is corroborated by a significantly more frequent consumption of fast food, fried food, and ultra-processed food. Increasingly demanding work schedules have led to the widespread use of the lunch box, the increased consumption of fast food, and frequent recourse to convenience and convenient foods as opposed to freshly cooked food and home-cooked meals, with undoubtedly negative effects on nutrition.

Between these two groups, we found no significant differences in the BMI, which could again be explained by the age of the sample, as noted above. This was a relatively young sample, with a mean age of 31.66 years for subjects living alone and 30.22 years for those living with others, and it is known that the prevalence of metabolic diseases increases with age, with a higher prevalence between 50 and 69 years for men and between 70 and 79 years for women [47].

On the other hand, sedentary habits and the time spent engaging in physical activity were found to be healthier for people living alone than for those living with others. We could attribute these results to the fact that living alone does not require social organization, which allows for a greater flexibility and more time to devote to different activities, including sports. The same healthy trend was observed for alcohol consumption, which was less frequent for people living alone compared to those living with others, and in water consumption, which was higher for the former.

Finally, using the same criteria for the healthy eating index of Norte and Ortiz [29], change is required for both groups, since, although we found that on many occasions the trend in the consumption habits of the cohabitation groups was healthier than in the groups of people living alone, we observed that they were below the recommendations for some aspects of healthy eating, such as weekly fish consumption. Change is also required in the sedentary behavior, as both groups spent between 7 and 9 h sitting, which is more than the 6 h a day that is considered healthy.

The main strength of this study lies in the sample size. On the other hand, its main limitation is that the results were obtained through an online survey; although this allows for the easy selection of a target population, it requires the availability of connection, which can undoubtedly lead to a response bias that we hoped to minimize by focusing the study on the population with the highest level of internet access. Another limitation is that we did not take into account the level of education in this first analysis.

## 5. Conclusions

The results obtained corroborate the data from the literature indicating that, in general, it is healthier to live and eat in company than in solitude, mainly in family company. People living alone have a worse healthy eating index, eat fish less frequently, and consume fast food, fried food, and ultra-processed food more frequently than those living with company. On the other hand, living in company seems to leave less time and less flexibility for physical activity, increasing the degree of sedentary lifestyles and decreasing the weekly time devoted to sports. All of the above seems to indicate that nutritional interventions should pay attention to living alone as a variable to be considered in future analyses.

## Figures and Tables

**Table 1:** Categorization of variables not included in the IASE.

Variable	Category	Score
Coffee consumption	2 glasses/cups a week or less	1
	2 glasses/cups per day or less	2
	Between 3 and 5 glasses per day	3
	More than 5 glasses/day	4
Water consumption	I do not drink water	1
	Less than 2 glasses a day	2
	Between 3 and 5 glasses a day	3
	More than 6 glasses per day	4
Consumption of fast food, fried, and ultra-processed dishes	Never	1
	Very seldom (2 times a month maximum)	2
	Once a week	3
	Several times a week	4
Fish consumption	Never or very seldom	1
	Between 1 and 2 times a week	2
	Three or more times a week	3
	Every day	4
Alcohol consumption	Never and once a month	1
	2 to 4 times a month	2
	2 to 3 times a week	3
	4 to 5 times a week and every day	4
Sitting time	Less than 7 h	1
	Between 7 and 9 h	2
	Between 9 and 11 h	3
	More than 11 h	4

**Table 2:** Sociodemographic characteristics of the sample.

	Mean ± SD or *n* (%)		
Male	3185 (17.7%)		
Female	14,784 (82.3%)		
Age (years)	30.3 ± 7.5		
Male age (years)	30.0 ± 7.7		
Female age (years)	30.4 ± 7.5		
	Total	18–30 years	>30 years
Age (*n* (%))		9750 (54.3%)	8219 (45.7%)
Male age (*n* (%))	3185 (17.7%)	1842 (10.3%)	1343 (7.5%)
Female age (*n* (%))	14,784 (82.3%)	7908 (44.0%)	6876 (38.2%)
Level of education			
Basic education	5746 (32.0%)	3533 (19.7%)	2213 (12.3%)
Higher education	12,223 (68%)	6217 (34.6%)	6006 (33.4%)
Income level			
Low	8396 (51.2%)	4910 (30%)	3486 (21.3%)
Medium–high	7993 (48.8%)	3616 (22.1%)	4677 (23.7%)
Place of residence			
Family home	13,025 (72.5%)	7024 (39.1%)	6001 (33.4%)
With relatives outside the home	383 (2.1%)	203 (1.1%)	180 (1.0%)
Alone in an apartment	1678 (9.3%)	915 (5.1%)	763 (4.1%)
Shared apartment	1876 (10.4%)	1045 (5.8%)	831 (4.6%)
Rented room	100 (0.6%)	52 (0.3%)	48 (0.3%)
Residence	169 (0.9%)	95 (0.5%)	74 (0.4%)
Other	738 (4.1%)	416 (2.3%)	322 (1.8%)

**Table 3:** Comparison of values differentiating between living at home and away from home.

	Mean ± SD		
	Resides at Family Home	Resides Away from Home	*p*-Value *
Body mass index (BMI), kg/m^2^	23.87 ± 4.74	23.35 ± 4.74	0.000
IASE score	54.20 ± 9.85	52.74 ± 10.04	0.000
Self-perceived health	3.82 ± 0.82	3.83 ±0.83	0.323
Sedentary lifestyle	2.42 ± 0.84	2.33 ± 0.86	0.000
Physical activity (min)	156.9 ± 178.7	171.4 ±181.1	0.000
Alcohol consumption	2.35 ± 0.80	2.18 ± 0.83	0.000
Water consumption	2.43 ± 0.63	2.44 ± 0.63	0.320
Coffee consumption	0.66 ± 0.70	0.71 ± 0.70	0.000
Fast-food consumption	1.55 ± 0.76	1.53 ± 0.75	0.195
Consumption of fried foods	1.71 ± 0.83	1.82 ± 0.80	0.000
Consumption of ultra-processed food	1.59 ± 0.95	1.62 ± 0.92	0.211
Fish consumption	0.81 ± 0.49	0.74 ± 0.51	0.000

* Mann–Whitney test.

**Table 4:** Comparison of values differentiating between residing alone and with company.

	Mean ± SD		
	Lives Alone	Lives Accompanied	*p*-Value *
Body mass index (BMI), kg/m^2^	23.82 ± 4.88	23.73 ± 4.65	0.459
IASE score	52.15 ± 10.44	54.07 ± 9.81	0.000
Self-perceived health	3.84 ± 0.83	3.83 ± 0.82	0.332
Sedentary lifestyle	2.34 ± 0.88	2.40 ± 0.84	0.003
Physical activity (min)	187.2 ±184.5	158.2 ±179.3	0.000
Alcohol consumption	2.17 ± 0.85	2.32 ± 0.80	0.000
Water consumption	2.47 ± 0.62	2.43 ± 0.63	0.009
Coffee consumption	0.79 ± 0.73	0.66 ± 0.69	0.000
Fast-food consumption	1.61 ± 0.76	1.53 ± 0.75	0.000
Consumption of fried foods	1.92 ± 0.78	1.71 ± 0.82	0.000
Consumption of ultra-processed food	1.68 ± 0.93	1.59 ± 0.94	0.000
Fish consumption	0.75 ± 0.51	0.80 ± 0.49	0.000

* Mann–Whitney test.

## Data Availability

Not applicable.
